# Sex differences in methylphenidate-induced dopamine increases in ventral striatum

**DOI:** 10.1038/s41380-021-01294-9

**Published:** 2021-10-27

**Authors:** Peter Manza, Ehsan Shokri-Kojori, Corinde E. Wiers, Danielle Kroll, Dana Feldman, Katherine McPherson, Erin Biesecker, Evan Dennis, Allison Johnson, Andrew Kelleher, Song Qu, Dardo Tomasi, Gene-Jack Wang, Nora D. Volkow

**Affiliations:** 1 National Institute on Alcohol Abuse and Alcoholism, National Institutes of Health, Bethesda, MD USA; 2 Department of Laboratory Medicine, National Institutes of Health, Bethesda, MD USA

**Keywords:** Psychology, Neuroscience

## Abstract

Sex differences in the prevalence of dopamine-related neuropsychiatric diseases and in the sensitivity to dopamine-boosting drugs such as stimulants is well recognized. Here we assessed whether there are sex differences in the brain dopamine system in humans that could contribute to these effects. We analyzed data from two independent [^11^C]raclopride PET brain imaging studies that measured methylphenidate-induced dopamine increases in the striatum using different routes of administration (Cohort A = oral 60 mg; Cohort B = intravenous 0.5 mg/kg; total *n* = 95; 65 male, 30 female), in blinded placebo-controlled designs. Females when compared to males reported stronger feeling of “drug effects” and showed significantly greater dopamine release in the ventral striatum (where nucleus accumbens is located) to both oral and intravenous methylphenidate. In contrast, there were no significant differences in methylphenidate-induced increases in dorsal striatum for either oral or intravenous administration nor were there differences in levels of methylphenidate in plasma. The greater dopamine increases with methylphenidate in ventral but not dorsal striatum in females compared to males suggests an enhanced sensitivity specific to the dopamine reward system that might underlie sex differences in the vulnerability to substance use disorders and to attention-deficit/hyperactivity disorder (ADHD).

## Introduction

Dopamine signaling in the brain is responsible for modulating functions critical for survival, including attention, memory, motor control, motivation, and reward-guided behaviors [1, 2]. Several lines of research point to sex-based differences in dopamine signaling, with consequences for health outcome disparities. Differential expression of genes in the sex chromosomes directly contributes to differences in midbrain dopaminergic signaling between men and women [3]. These are thought to manifest in differences in brain structure and function that emerge in childhood, impacting behavior and vulnerability to neuropsychiatric illnesses including those associated with aberrant dopaminergic function [4]. For instance, men are about 2.5 times more likely to be diagnosed with attention-deficit/hyperactivity disorder (ADHD) than women, whereas women are almost twice as likely to be diagnosed with a mood disorder than men [e.g., 5, 6].

Similarly, the prevalence of most substance use disorders (SUD) including that for stimulants is higher for males than females [7]. However, differences in prevalence are driven in part by environmental factors that make women less likely to be exposed to drugs than men, particularly in adolescents and young adults [8]. Moreover, the sensitivity to stimulant drugs appears to be higher for women than for men. Women experience more severe symptoms of cocaine use disorder than men: they transition from occasional cocaine taking to addiction more rapidly, exhibit greater cue-induced craving and withdrawal symptoms, and have poorer treatment outcomes [7, 9, 10]. Similarly, in the case of methamphetamine, women take more drugs and transition from occasional use to dependence faster than men [11]. Studies in human laboratory settings further suggest that women are more sensitive to the psychomotor and subjective behavioral effects of methamphetamine than men [12]. Preclinical studies have corroborated significant sex differences in the response to stimulant drugs including locomotor and rewarding effects [13, 14]. These findings underscore the need to better understand sex differences in the brain’s responses to dopamine-enhancing drugs in humans that might help clarify sex differences in vulnerability to SUD and other neuropsychiatric disorders that involve the dopaminergic system (e.g., ADHD, schizophrenia, mood disorders, and Parkinson’s disease).

Since increases in dopamine in the nucleus accumbens (NAc) are associated with the rewarding effects of addictive drugs, a first step is to examine if women and men differ in their dopaminergic response to drugs in this striatal brain region. Positron emission tomography (PET) has been the primary tool used to measure drug-induced dopamine increases in the human brain. Studies rely on a PET radiotracer ligand that competes with dopamine for binding to dopamine receptors (e.g., [^11^C]raclopride, which binds to D_2_ and D_3_ [D_2/3_] receptors) and compare its binding after placebo and after a drug or a behavioral challenge. If the challenge increases dopamine levels, the endogenous dopamine occupies D_2/3_ receptors, decreasing their availability, and hence [^11^C]raclopride binding is reduced (the decrease in receptor availability is a marker of ‘dopamine release’). To our knowledge, three PET studies have examined sex differences in striatal dopamine release using the stimulant amphetamine as a challenge and [^11^C]raclopride or [^18^F]fallypride as D_2/3_ receptor radiotracers. One study using [^11^C]raclopride reported greater striatal dopamine release after intravenous amphetamine (0.3 mg/kg) in men than women (28 men, 15 women; 18–29 years) [15]; a smaller study using [^18^F]fallypride reported higher dopamine release in pallidum after oral ampethamine (0.43 mg/kg) in women than men (seven men, six women; 21–32 years of age) [16]; and another [^18^F]fallypride study did not observe consistent sex differences after oral amphetamine (0.43 mg/kg) (Two independent cohorts total: 37 males, 39 females; aged 20–65) [17]. Hence, it is currently unresolved whether there are sex differences in dopamine release in humans. Here we assessed sex differences using methylphenidate, a drug that increases dopamine by blocking its reuptake into dopamine terminals and whose effects are dependent on dopamine neuronal activity (in contrast to amphetamine, which increases dopamine by directly releasing it from the terminal independent of dopamine neuronal firing) [18]. We also assessed sex differences after oral and intravenous administration since these two routes of administration have distinct reinforcing effects [19]. A previous study from our lab found that an intravenous methylphenidate challenge increased brain glucose metabolism in healthy females more than in males, particularly in cerebellum and midbrain, which we interpreted as indicative of elevated dopaminergic transmission in females [20].

Here we tested for potential sex differences in drug-induced dopamine increases, focusing on the NAc, a ventral striatal region critical for processing the rewarding effects of drugs [21], and contrasted it with the dorsal striatum, which is predominantly involved with motor and cognitive processes [22]. We hypothesized that women compared to men would show higher dopamine increases in response to methylphenidate in NAc but not in the dorsal striatum and would report greater subjective effects to the methylphenidate challenge since women appear to be more sensitive to cocaine, another drug that like methylphenidate increases dopamine by blocking the dopamine transporter [23].

## Materials and methods

### Participants

We report on results from two independent cohorts, referred to as Cohort A (60 mg oral dose of methylphenidate) and Cohort B (0.5 mg/kg intravenous dose of methylphenidate). All participants provided written informed consent. The Institutional Review Board committee of the National Institutes of Health (Cohort A) and Stony Brook University (Cohort B) approved the studies. For detailed characteristics of Cohort A (*n* = 32; 20 male, 12 female, age 22–64) and Cohort B (*n* = 63, 45 male, 18 female; age 19–50), see Table 1. Participants were excluded if they had a history of substance abuse or dependence (other than nicotine) or a history of psychiatric disorder, neurological disease, medical conditions that may alter cerebral function (i.e., cardiovascular, endocrinological, oncological, or autoimmune diseases), current use of prescribed or over-the-counter medications, and/or head trauma with loss of consciousness of >30 min.

**Table 1:** Demographics and characteristics for both cohorts.

	Mean (SD)	Mean (SD)	*t, p*
Cohort 1 (Oral 60 mg)	Males (*n* = 20)	Females (*n* = 12)	
Age	40.844 (12.135)	44.983 (12.452)	0.925, 0.362
BMI	27.735 (3.250)	27.608 (5.261)	−0.085, 0.933
Edu	15.650 (1.531)	15.75 (2.006)	0.159, 0.875
% Caucasian	50	33.33	
% African American	45	50	
Cohort 2 (IV 0.5 mg/kg)	Males (*n* = 45)	Females (*n* = 18)	
Age	35.999 (8.273)	30.672 (7.763)	−2.344, 0.022
BMI	25.634 (2.855)	24.513 (2.635)	−1.438, 0.156
Edu	14.534 (2.291)	14.833 (2.282)	0.467, 0.642
% Caucasian	35.56	11.11	
% African American	51.11	44.44	

*BMI* Body-Mass Index, *Edu* Years of Education.

### PET acquisition and drug administration

Cohort A. [^11^C]raclopride scans were performed on one of two scanners: a high-resolution research tomography (HRRT) scanner (*n* = 16; 7 female; Siemens AG; Germany) or a Biograph PET/CT scanner (*n* = 16; 5 female; Siemens AG; Germany). The methods for correcting differences between scanners are described in the *PET analysis* section below. Scans were conducted on two separate days: once 1 h after administration of an oral placebo pill (to assess baseline dopamine D_2/3_ receptor availability) and once 1 h after administration of 60 mg oral methylphenidate (single-blind; counterbalanced session order). All scans were conducted at the same time of day (1 PM) and in the same scanner for a given subject. Emission scans were started immediately after injection of 10 mCi (specific activity ≥500 mCi/µmol at end of bombardment). Twenty-two dynamic emission scans were obtained from time of injection up to 60 min after and arterial sampling was used to quantify total carbon-11 and unchanged [^11^C]raclopride in plasma. Dynamic emission scan images were evaluated before analyses to ensure that motion artifacts or misplacements were not included.

Cohort B. [^11^C]raclopride scans were acquired on a Siemens HR + scanner and some of the data was reported previously for different study purposes [24–26]. Scans were conducted on two separate days: once 2 min after 3 ml intravenous saline placebo (to assess baseline dopamine D_2/3_ receptor availability) and once 2 min after 0.5 mg/kg intravenous methylphenidate (single-blind; counterbalanced session order). Details on this PET scanning protocol have been previously described [25]. In short, emission scans were started immediately after injection of 4–8 mCi (specific activity 500–1500 mCi/µmol at end of bombardment). Twenty-one dynamic emission scans were obtained from time of injection for a total of 60 min and arterial sampling was used to quantify total carbon-11 and unchanged [^11^C]raclopride in plasma. In addition, the dynamic emission scan images were evaluated before analyses to ensure that any motion artifacts or misplacements were not included.

### PET analysis

Cohort A. PET images were coregistered to high-resolution MRI scans: a T1 (3D MP-RAGE; TR/TE = 2400/2.24 ms) and T2 (SPACE; TR/TE = 3200/564 ms) image each with 0.8 mm isotropic voxels, acquired on a 3.0T Magnetom Prisma scanner (Siemens Medical Solutions USA, Inc., Malvern, PA) with a 32-channel head coil. We used the minimal preprocessing pipelines of the Human Connectome Project for the spatial normalization to the stereotactic MNI space of the structural and PET scans [27]. Differences in geometry and PSF between cameras (PET/CT = 4 mm PSF; HRRT = 2.7 mm PSF) originated systematic voxelwise differences in signal intensity between PET/CT and HRRT images. To correct for these scanner-specific scaling effects and harmonize the data we used a voxelwise approach based on grand-mean scaling. Specifically, the scaling matrix *M*(*x,y,z*) was estimated from *m* = 16 PET/CT images, $\beta _i(x,y,z)$, and *n* = 16 HRRT images, $\alpha _i(x,y,z)$, in MNI space using:

$$M\left( {x,y,z} \right) = \frac{{m\mathop {\sum }\nolimits_{i = 1}^n \alpha _i(x,y,z)}}{{n\mathop {\sum }\nolimits_{i = 1}^m \beta _i(x,y,z)}}$$

Successively, the PET/CT images in MNI space were corrected to match the average distribution of signal intensity of the HRRT images using:

$$\beta ^c_i\left( {x,y,z} \right) = M\left( {x,y,z} \right) \ast \beta _i\left( {x,y,z} \right)$$

FreeSurfer version 5.3.0 (http://surfer.nmr.mgh.harvard.edu) was used to automatically segment the anatomical MRI scans using the Desikan atlas [28], which provided bilateral NAc, caudate/putamen, and cerebellum regions of interest (ROIs).

Cohort B. Because we did not have structural MRI scans for this cohort, we calculated the non-displaceable binding potential (BPnd) values for the hand-drawn region of interest (ROI) in NAc, caudate, and putamen and used a cerebellar ROI to assess nonspecific binding using a procedure previously described [29]. Bilateral ROIs were drawn directly in an averaged emission image (summation of images obtained between 10 and 60 min). NAc, caudate, and putamen ROIs were obtained from three sequential axial planes where the ROIs were most visible and had the same size and shape across subjects (0.8, 2.2, and 2.2 mm^3^, respectively). For the cerebellum, we averaged the values obtained from circular ROIs in the left and right cerebellum (16 mm^3^) in three contiguous axial planes positioned within 1.0 and 1.7 cm above the canthomeatal line. ROI values were computed using the weighted average for left and right regions from the different slices where the regions were obtained.

Both cohorts. Time–activity curves in the dorsal striatum (caudate and putamen), NAc, and cerebellum were used to obtain the distribution volume ratios (DVR) using a Logan reference tissue model [30, 31]. The NAc-to-cerebellum and the dorsal striatum-to-cerebellum DVRs correspond to BPnd+1, which was used to quantify D_2/3_ receptor availability. We averaged the values for caudate and putamen to create one ‘dorsal striatum’ ROI, since caudate and putamen BPnd are highly correlated with one another (across all participants in both cohorts, *r* ≈ 0.9).

### Behavioral and heart rate data acquisition

To assess subjective drug effects, participants in both cohorts were asked by experimenter questions from the ‘Drug Effects Questionnaire’ throughout the experiment, starting 5 min before drug administration until the end of the PET scan. Participants were generally queried every 1–5 min, and at no time was there more than 10 min between subsequent queries. For the current manuscript, we selected the only two measures from the questionnaire for which we had data in common between the two cohorts: (1) “Do you feel any drug effects?” and (2) “Do you feel high?”. Participants were asked to rate on a scale of 1–10, with 1 meaning “not at all” and 10 meaning “maximum”. During the same time points, we also collected heart rate using an electrocardiogram to assess cardiovascular responses to the drug.

### Plasma methylphenidate concentrations

To determine if any sex differences in dopamine release might be attributed to sex differences in drug availability, we acquired blood samples in Cohort A at −15, 30, 60, 90, 120, 180, and 300 min relative to drug administration; and in Cohort B at 10, 25, 40, and 55 min post drug administration. Methylphenidate concentration in plasma was measured using UPLC-MS/MS (Cohort A) and capillary GC/MS (Cohort B).

### Menstrual cycle and sex hormone analyses

To determine if any sex differences in dopamine release might be driven by specific phases of the menstrual cycle, we also measured plasma levels of sex hormones: follicular stimulating hormone, luteinizing hormone, progresterone, estradiol, and the progresterone:estradiol ratio. These were analyzed using the first (pre-drug) blood sample on the methylphenidate study day, using a chemiluminescent microparticle immunoassay (CMIA).

### Statistical analyses

Analyses were performed in R version 3.6.2 and in GraphPad Prism version 8.0.1. To test for sex differences in dopamine release, we constructed linear models, where sex was the predictor variable, age and BMI were covariates, and the outcome variable was dopamine release. The same analyses were performed in cohort A and cohort B. Analyses were performed in NAc and in dorsal striatum to assess if effects were specific to NAc.

We also tested for sex differences in subjective drug effects (i.e., ‘high’ and ‘feel effects’) as well as differences in heart rate and plasma concentrations using a sex-by-time repeated measures ANOVA, controlling for age and BMI. To get comparable timepoints for ‘high’ and ‘feel effects’ ratings in both cohorts, which had different acquisition times due to the differing drug administration routes, we took data from every 10 min starting from PET acquisition to the end of the scan (i.e., 0:10:60 min). Since healthy adults often do not report feeling any subjective effects from oral methylphenidate [32], we also performed a follow-up chi-squared analysis on binarized ratings, to see if there were sex differences in perception ratings (i.e., rating all 1’s was considered ‘not feeling effects’ and any rating >1 was considered ‘feeling effects’). The sex-by-time repeated measures ANOVA for plasma methylphenidate was conducted using the four time points corresponding to the period of maximal drug efficacy (Cohort A: 30, 60, 90, and 120-min time points; Cohort B: 10, 25, 40, and 55-min time points) to confirm that any sex differences were not due to differences in drug bioavailability. In a separate analysis, including the later timepoints collected in cohort A (180 and 240 min time points) did not alter the conclusions. Note that due to some data being unavailable, plasma data for Cohort A had a final sample of *n* = 29, 18 M/11 F; and that subjective effects and plasma data for Cohort B had a final sample of *n* = 42, 27 M/15 F. All other analyses were conducted in the full sample presented in Table 1.

Finally, to assess whether any sex differences in dopamine release might vary as a function of menstrual cycle stage, we performed follow-up analyses that included measures of cycle stage as covariates. To estimate cycle phase we used both self-reports (recorded days since last menstruation) and plasma levels of sex hormones. For self-report, cycle phase was characterized based on a recent review with recommendations [33]: 1–12 days since last menses = follicular; 13–15 days = ovulation; 16–28 days = luteal; or post-menopausal. This data was only available for Cohort A.

## Results

**Fig. 1:**
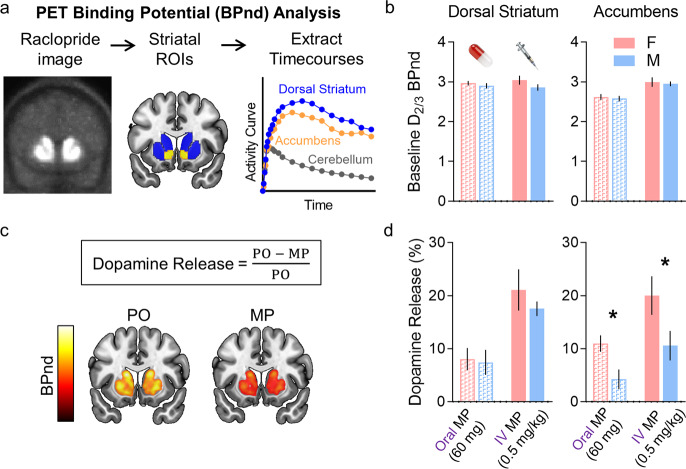
PET analysis and results. **a** PET analysis flow. Using template regions of interest (ROIs), we extracted dorsal striatal (caudate and putamen) and nucleus accumbens time courses from participant [^11^C]raclopride images. Dopamine D_2/3_ receptor non-displaceable binding potential (BPnd) was calculated for each region based on the activity relative to the cerebellum reference region, using the Logan model [30]. **b** There were no significant sex differences in ‘baseline’, i.e. placebo (PO) D_2/3_ BPnd. **c** ‘Dopamine Release’ is measured based on the drop in raclopride signal following methylphenidate (MP) administration relative to PO. **d** In both cohorts, females (F) showed significantly greater dopamine release to the MP challenge than males (M). For scatter plots showing the individual BPnd data points for PO and MP scans, see Supplementary Figure S1. Note: *=*p* < 0.05.

We constructed linear models to determine if sex was significantly associated with NAc methylphenidate-induced dopamine increases in NAc, controlling for age and BMI. We first ensured that there were no significant sex differences in baseline D_2/3_ BPnd, neither in dorsal striatum (Cohort A: *t* = 1.124, *p* = 0.270; Cohort B: *t* = 0.301, *p* = 0.764), nor in NAc (Cohort A: *t* = 0.763, *p* = 0.452; Cohort B: *t* = 0.357, *p* = 0.722; Fig. 1b). As hypothesized, we showed significant sex differences in dopamine release in NAc; specifically, females showed significantly higher dopamine release than males (Cohort A: *t* = 2.483, *p* = 0.019; Cohort B: *t* = 2.009, *p* = 0.049; Fig. 1d, right). Since in Cohort A the standard dose of 60 mg was not body weight-adjusted, we also tested this model with weight instead of BMI. Results were essentially unchanged: sex differences in NAc dopamine release remained significant (*t* = 2.511; *p* = 0.018). The associations between NAc dopamine release and age (Cohort A: *t* = −0.115, *p* = 0.908; Cohort B: *t* = 0.470, *p* = 0.640) and between NAc dopamine release and BMI (Cohort A: *t* = 0.635, *p* = 0.531; Cohort B: *t* = 0.216, *p* = 0.830) were not significant. In contrast, there were no significant sex differences in dopamine release in the dorsal striatum (Cohort A: *t* = 0.260, *p* = 0.797; Cohort B: *t* = 0.834, *p* = 0.408, Fig. 1d, left). Variances in NAc dopamine release were not significantly different between males and females (Cohort A: *F*_(19,11)_ = 2.385, *p* = 0.142; Cohort B: *F*_(44,17)_ = 1.451, *p* = 0.409).

**Fig. 2:**
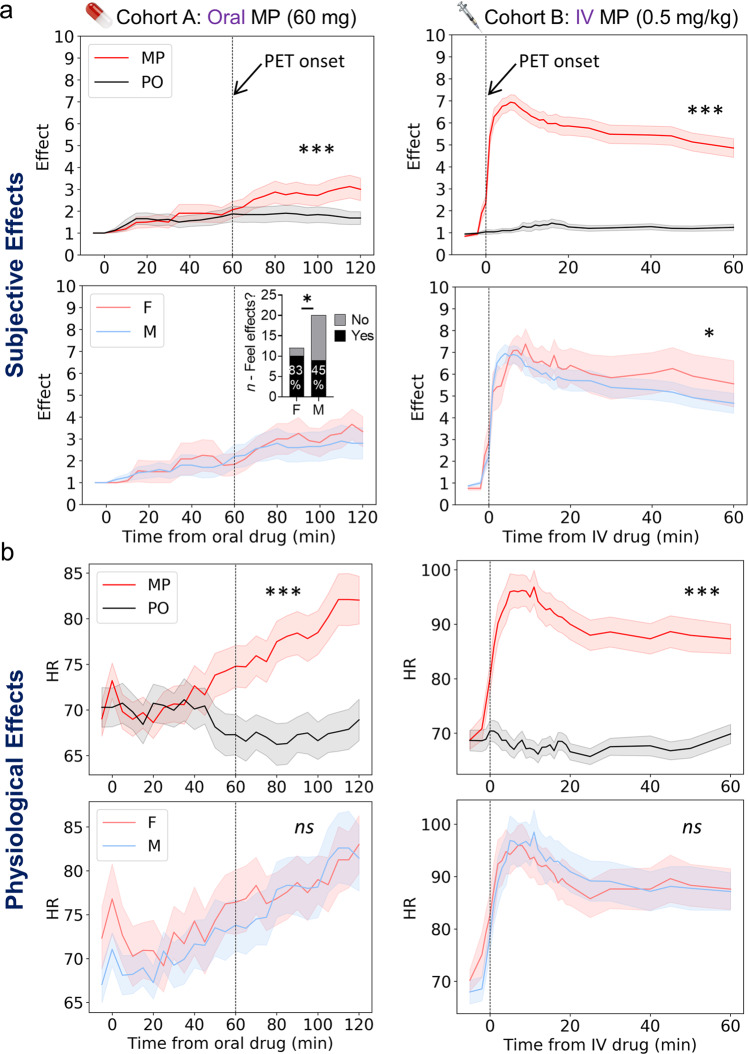
Subjective and physiological effects of methylphenidate in males and females. Subjective (self-report of “Feel Drug”) (**a**) and physiological (heart rate) (**b**) effects of methylphenidate (MP) versus placebo (PO), and sex effects for the MP session. Females (F) reported greater subjective effects to oral and intravenous (IV) MP than males (M; 2nd row), however, there were no significant sex differences in heart rate (HR; 4th row). Note: *=*p* < 0.05; ***=*p* < 0.001; ns not significant.

We also tested whether behavioral responses to methylphenidate varied significantly by sex, using a sex-by-time repeated measures ANOVA. In both cohorts, as expected, overall self-reported “feel drug effects” and the heart rate was higher in the methylphenidate compared to placebo condition (*p*’s < 0.001; Fig. 2). In Cohort A, there were no significant main effects of sex nor sex-by-time interactions for ‘feel drug effects’ or ‘high’ (for complete results of ANOVA tables, see Supplementary Table S1). However, there were strong floor effects in this data, as nearly half of the participants reported no effects for all timepoints (13 out of 32). To address this, we took the max rating of all timepoints and binarized the data (max score >1: ‘feel effects’, max score = 1: ‘did not feel effects’). Using this approach, 10 out of 12 females (83.3%) reported ‘feeling drug effects’ versus only nine out of 20 males (45%); χ^2^ = 4.579, *p* = 0.033; Fig. 2a, inset. This effect was marginally significant for ‘high’ ratings: seven out of 12 females (58%) reported ‘feeling high’ versus seven out of 20 males (35%); χ^2^ = 3.791, *p* = 0.051 (not shown). In Cohort B, the main effect of sex was significant for ‘feel effects’: *F*_(1,334)_ = 3.875, *p* = 0.049, again with females reporting greater effects than males. However, the main effect of sex was not significant for ‘high’ ratings *F*_(1,334)_ = 0.020, *p* = 0.888. There were no significant sex-by-time effects for either measure (Supplementary Table S1). We did not binarize the data for this cohort because every participant reported ‘feeling effects’ or ‘high’ from the intravenous dose. Finally, heart rate, which was significantly increased by methylphenidate did not vary as a function of sex in either cohort: neither the main effect of sex nor the sex-by-time interaction were significant (Fig. 2b).

Differences in methylphenidate plasma levels did not drive the sex differences in dopamine release in NAc nor the subjective drug effects. The sex-by-time interaction was not significant: Cohort A: *F*_(3,102)_ = 0.317, *p* = 0.813; Cohort B: *F*_(3,154)_ = 0.122, *p* = 0.947. Moreover the main effect of sex was not significant in Cohort A: *F*_(1,102)_ = 0.679, *p* = 0.412; and for Cohort B, the concentration of methylphenidate in plasma was significantly higher in men than women (repeated measures ANOVA, main effect of sex: *F*_(1,154)_ = 4.699, *p* = 0.032; post-hoc Tukey’s HSD test: Male > Female, mean difference = 12.303, 95% CI = [1.091 23.514], *p* = 0.032). For full ANOVA results see Supplementary Table S2; Supplementary Figure S2.

Likewise, differences in menstrual cycle phase did not appear to play a major role in the sex differences in dopamine release in NAc. Follow-up analyses that included the following additional covariates: plasma levels of progresterone (*t* = −2.387, *p* = 0.027), follicular stimulating hormone (*t* = −2.637, *p* = 0.015), and luteinizing hormone (*t* = −2.349, *p* = 0.029), as well as estimated cycle phase based on self-reported days since last menstruation (*F*_(1,25)_ = 4.336, *p* = 0.047), all remained significant for sex differences in NAc dopamine release (Cohort A). Meanwhile, in all of these analyses, the effect of the sex hormone quantities and self-reported menstrual phase were not significant (all *p*’s > 0.25). Due to insufficient plasma sample volume for some participants, data on estradiol was only available for *n* = 17 (seven female) participants, and we were thus insufficiently powered to do the same analysis with estradiol and the progresterone:estradiol ratio. However, the preliminary data suggests that these markers are also not strongly associated with methylphenidate-induced dopamine release in NAc. For visualization of individual datapoints showing the (lack of) association between all hormones and NAc dopamine release, see scatter plots in Supplementary Figure S3.

## Discussion

Here we find that females compared to males showed larger dopamine increases in ventral striatum (NAc) when challenged with methylphenidate whether it was given orally or intravenously whereas we found no differences in the dorsal striatum. These results provide evidence of sex differences in sensitivity of the dopamine reward system with females showing greater sensitivity than males. The replication of sex differences in dopamine release in NAc and of no sex differences in the dorsal striatum in two independent cohorts supports the robustness of the findings and indicate that the sex differences were specific for NAc.

Females reported feeling greater “drug effects” (though not stronger ‘high’) than males and showed higher dopamine release in NAc than males to methylphenidate, for both a fixed oral dose (60 mg) and an intravenous weight-adjusted dose (0.5 mg/kg). These sex differences remained significant after controlling for BMI, which has known associations with striatal D_2/3_ receptor availability [34, 35] and with dopamine release in NAc in response to a high-calorie beverage [36]. Therefore, our data indicate that sex differences in BMI did not account for the sex differences in dopamine release in NAc. Further, for the intravenous study, females showed lower methylphenidate plasma concentration than men, suggesting that the higher dopamine increases in females were not due to having greater drug plasma concentrations than males. The specificity of sex differences in dopamine release in NAc but not in dorsal striatum also indicates that sex differences in drug bioavailability are not driving the findings, for they would have equally influenced ventral and dorsal striatum. Thus, our findings in NAc are likely to reflect sex differences in the function of the mesoaccumbens dopamine reward pathway. Indeed, preclinical studies have documented significant sex differences in the mesoaccumbens dopamine pathway [37] including a larger proportion of VTA dopamine neurons in female than in male rats [38] as well as greater dopamine release in the caudate nucleus following electrical stimulation of the VTA in females than males regardless of stage in the estrous cycle [39]. Preclinical studies have also documented modulation of VTA dopamine neuronal activity by estrogens in females, which makes them more vulnerable to drug-taking and escalation [40].

The higher subjective response to methylphenidate in females than in males is consistent with findings from a study in overweight/obese women with and without binge eating disorder who reported stronger drug effects in response to methylphenidate than men [41]. They may also pertain to reported sex differences in the efficacy of methylphenidate for the treatment of ADHD in children, which showed that females compared to males had greater symptom reduction in the first few hours during peak drug efficacy [42]. Prescription of stimulants for ADHD and stimulant misuse (particularly for amphetamines) has risen rapidly in the past decade in the United States among adults, and more women aged 30 and over are now using stimulants than men [43]. Therefore, it is important to understand whether stimulant dosage might need to be adjusted based on sex to maximize therapeutic efficacy and minimize its potential diversion and misuse. Nevertheless, the sex differences in subjective effects, while significant, were of modest effect size. Changes in brain function are not always sufficient to produce changes in behavior; indeed, it is very likely that some buffering happens and that quite large changes in brain function are needed to produce measurable changes in behavior. This has been documented extensively, and one prominent example comes from the aging literature: there are many (often quite large) changes in brain structure and function that occur throughout the lifespan but do not result in measurable changes in behavior (often termed brain ‘resilience’ or ‘maintenance’, and recently reviewed by [44]). From this perspective, the fact that we still observed significant behavioral differences in both studies speaks to the strength of this effect.

Our results differ from the three prior PET studies that found no consistent sex differences in dopamine release when administering amphetamine [15–17]. Two of these studies used [^18^F]fallypride [16, 17] whereas one of them, like our study, used [^11^C]raclopride and since these two radiotracers differ in their sensitivity to striatal and extra striatal dopamine this could have contributed to the differences across studies. More importantly, prior studies used amphetamine, whereas we used methylphenidate, and the differences in their mechanism of action could account for the discrepancies. Methylphenidate’s primary mechanism of action is to block dopamine transporters, whereas amphetamine directly releases dopamine from cell vescicles via deacidification into the synaptic cleft via reverse transport through dopamine transporters [18, 45, 46]. Therefore, amphetamine increases synaptic dopamine independent of cell firing, whereas methylphenidate increases synaptic dopamine to a degree that depends on action potential firing rate to elicit vesicular release. Since VTA dopamine neuronal firing is highest when levels of circulating estrogen peak [47] these fluctuations would have affected the effects of methylphenidate more than those of amphetamine. Further, since estrogen modulates the reactivity of VTA dopamine neurons to drug rewards [14] the time during the menstrual cycle at which the PET measures were obtained could have also contributed to the discrepancies between the studies. The [^18^F]fallypride study that did not observe consistent sex differences included 39 females of whom 18 were on hormonal birth control, 10 were postmenopausal and 11 were studied during the luteal phase [17] and the other included only six young women but the time of the menstrual cycle was not specified [16]. The [^11^C]raclopride study that reported greater increases in males than females included 15 young women, six of whom were studied in the luteal phase and nine in the follicular phase [15]. While Munro and colleagues did not see differences in amphetamine-induced dopamine increases between women studied during the luteal and the follicular phase, D_2/3_ receptor baseline availability was lower for women in the luteal phase than in the follicular phase (though this association with baseline receptor availability was not observed more recently in a larger cohort: [48]). Nevertheless, since this appears to be the first study to explicitly examine sex differences in dopamine release to a methylphenidate challenge, the current findings warrant replication.

In our study, we showed that even though Cohort B males had a higher level of methylphenidate in plasma than females, they had smaller dopamine increases with methylphenidate. This could reflect lower activity of VTA dopamine neurons in males than in females resulting in lower dopamine increases when given methylphenidate despite their having higher plasma levels [49]. Nonetheless, a preclinical study reported higher brain concentrations of methylphenidate in females than in male rats, which the authors speculated could reflect increased transport across the blood–brain barrier or reduced methylphenidate metabolism [50] and so while unlikely we cannot completely rule out potential sex differences in methylphenidate content in brain. It is currently not feasible to measure metabolism of methylphenidate in the human brain, but as new technologies emerge it might be possible to measure this and test whether there are sex differences in methylphenidate’s metabolism between ventral and dorsal striatum. Regardless, across most strains of rats [51–53] and in humans [20, 41], females appear to be more sensitive to the subjective and behavioral effects of methylphenidate. Here we show evidence for this phenomenon in the human brain.

To the extent that an enhanced sensitivity of the dopamine reward system to drugs would strengthen conditioned responses [54] our findings could explain the greater vulnerability of women to transition from drug-taking into addiction than men (the ‘telescoping’ effect; [37]). Indeed, clinical and preclinical studies have reported greater conditioning to drug cues in females than in males [10]. Our findings of greater reactivity of the mesoaccumbens dopamine system in females could also pertain to the greater sensitivity of women to stress, which is mediated in part by differences in VTA dopamine neuronal reactivity [55].

Limitations. In Cohort A, PET data were collected on two different scanners, which could confound findings. However, there were no significant differences in participant demographics based on the scanner used; we used methods to correct for any average differences in scanners; and our primary findings were replicated in Cohort B, where all PET data were collected on the same scanner. In addition, our data were collected in a sample with a relatively wide age range (19–64), which could impact the findings since age is strongly associated with dopamine signaling [56], although results remained significant after statistically controlling for age. Finally, with PET and [^11^C]raclopride we cannot determine where the location of the DA increases from methylphenidate occurred (synaptic or extrasynaptic) [57].

In summary, we showed that there are sex differences in methylphenidate-induced dopamine increases in NAc that might underlie the greater vulnerability of females to drug conditioning and to addiction. These findings also have implications for sex differences in other neuropsychiatric conditions associated with aberrant dopamine signaling in mesoaccumbens pathways, including ADHD and depression.

## Supplementary information

Supplement

## Data Availability

Summary data and code used to produce these results will be made available upon reasonable request.
