# Supplementary material for: Sex differences in methylphenidate-induced dopamine increases in ventral striatum
Source: Mol Psychiatry. 2021 Oct 27;27(2):939–46. doi: 10.1038/s41380-021-01294-9 (PMC9043036; doi:10.1038/s41380-021-01294-9)

**Supplementary Information: Sex differences in methylphenidate-induced dopamine increases in ventral striatumTable S1**. Subjective (‘Feel Drug Effects’ and ‘High’) and physiological (heart rate) effects during the methylphenidate session; repeated measures ANOVA (Time × Sex, controlling for Age and BMI).

|  | COHORT A (60 mg oral) | | | | |  | COHORT B (0.5 mg/kg IV) | | | | |
| --- | --- | --- | --- | --- | --- | --- | --- | --- | --- | --- | --- |
|  | Df | Sum of Squares | Mean Square | *F* value | *p* value |  | Df | Sum of Squares | Mean Square | *F* value | *p* value |
| \|  \| FEEL DRUG EFFECTS \| \| --- \| --- \| | | | | | | | | | | | |
| Time | 6 | 21.8 | 3.64 | 0.567 | 0.757 |  | 6 | 516.8 | 86.1 | 11.635 | 7.69E-12 |
| Sex | 1 | 2.4 | 2.41 | 0.375 | 0.541 |  | 1 | 28.7 | 28.7 | 3.875 | 0.0498 |
| Age | 1 | 202.2 | 202.18 | 31.476 | 6.39E-08 |  | 1 | 330.6 | 330.6 | 44.654 | 9.86E-11 |
| BMI | 1 | 7 | 6.97 | 1.086 | 0.299 |  | 1 | 0.2 | 0.2 | 0.028 | 0.8673 |
| Time × Sex | 6 | 5.4 | 0.9 | 0.14 | 0.991 |  | 6 | 1.8 | 0.3 | 0.041 | 0.9997 |
|  |  |  |  |  |  |  |  |  |  |  |  |
|  | HIGH | | | | | | | | | | |
| Time | 6 | 10.4 | 1.74 | 0.277 | 0.9473 |  | 6 | 531.4 | 88.57 | 10.532 | 1.04E-10 |
| Sex | 1 | 0.1 | 0.09 | 0.014 | 0.9069 |  | 1 | 0.2 | 0.17 | 0.02 | 0.888 |
| Age | 1 | 191.2 | 191.23 | 30.502 | 9.89E-08 |  | 1 | 198.7 | 198.69 | 23.627 | 1.80E-06 |
| BMI | 1 | 35.9 | 35.93 | 5.732 | 0.0175 |  | 1 | 1.3 | 1.26 | 0.15 | 0.699 |
| Time × Sex | 6 | 2.7 | 0.46 | 0.073 | 0.9985 |  | 6 | 12.8 | 2.14 | 0.254 | 0.957 |
|  |  |  |  |  |  |  |  |  |  |  |  |
|  | HEART RATE | | | | | | | | | | |
| Time | 6 | 1498 | 249.6 | 1.379 | 0.22459 |  | 6 | 3507 | 585 | 1.829 | 0.0912 |
| Sex | 1 | 51 | 50.5 | 0.279 | 0.59787 |  | 6 | 3507 | 585 | 1.829 | 0.0912 |
| Age | 1 | 537 | 537.3 | 2.967 | 0.08644 |  | 1 | 124 | 124 | 0.389 | 0.5329 |
| BMI | 1 | 2226 | 2226 | 12.294 | 0.00056 |  | 1 | 7467 | 7467 | 23.368 | 1.72E-06 |
| Time × Sex | 6 | 157 | 26.1 | 0.144 | 0.99 |  | 1 | 1014 | 1014 | 3.175 | 0.0753 |
|  |  |  |  |  |  |  |  |  |  |  |  |

**Table S2**. Plasma concentrations of methylphenidate; repeated measures ANOVA (Time × Sex, controlling for Age and BMI). To make the analysis comparable between the cohorts, which had different routes of administration/dose and therefore different pharmacokinetic profiles, we used the four timepoints corresponding to the period of maximal drug efficacy (Cohort A: 30, 60, 90, and 120-minute timepoints; Cohort B: 10, 25, 40, and 55-minute timepoints). In a separate analysis, including the later timepoints collected in cohort A (180 and 240 min timepoints) did not alter the conclusions. For individual data points at each time point see **Figure S2**. see Note; in Cohort B the significant effect of sex was in the direction: Male > Female; Tukey’s HSD test: mean difference = 12.303, 95% CI = [1.091 23.514]. Therefore, higher accumbens dopamine release in females compared to males is not likely due to differences in drug bioavailability.

|  | COHORT A (60 mg oral) | | | | | COHORT B (0.5 mg/kg IV) | | | | |
| --- | --- | --- | --- | --- | --- | --- | --- | --- | --- | --- |
|  | Df | Sum of Squares | Mean Square | *F* value | *p* value | Df | Sum of Squares | Mean Square | *F* value | *p* value |
| Time | 3 | 2790 | 929.9 | 8.763 | 3.2e-05 | 3 | 160116 | 53372 | 43.549 | <2e-16 |
| Sex | 1 | 72 | 72.1 | 0.679 | 0.4118 | 1 | 5759 | 5759 | 4.699 | 0.0317 |
| Age | 1 | 8 | 7.5 | 0.071 | 0.7905 | 1 | 4805 | 4805 | 3.92 | 0.0495 |
| BMI | 1 | 1235 | 1234.7 | 11.636 | 0.0009 | 1 | 8054 | 8054 | 6.572 | 0.0113 |
| Time × Sex | 3 | 101 | 33.6 | 0.317 | 0.8132 | 3 | 448 | 149 | 0.122 | 0.9471 |

**Figure S1**. Scatterplot of non-displaceable binding potential (BPnd) for the placebo (PO) versus methylphenidate (MP) sessions.


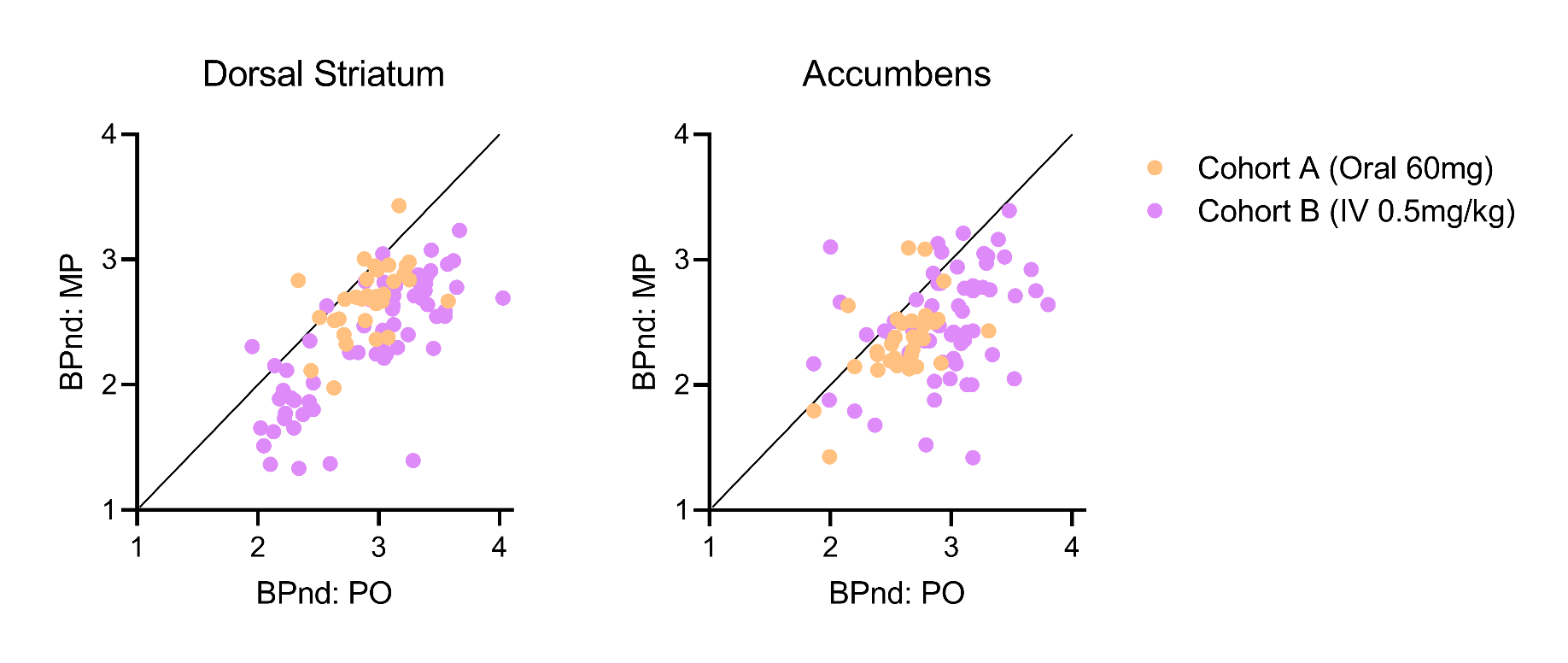


**Figure S2**. Plasma concentrations of methylphenidate (MP) for females (F) and males (M). Analyses for the main effect of sex and the time-by-sex interaction demonstrated that females did not show significantly higher plasma concentrations of methylphenidate.


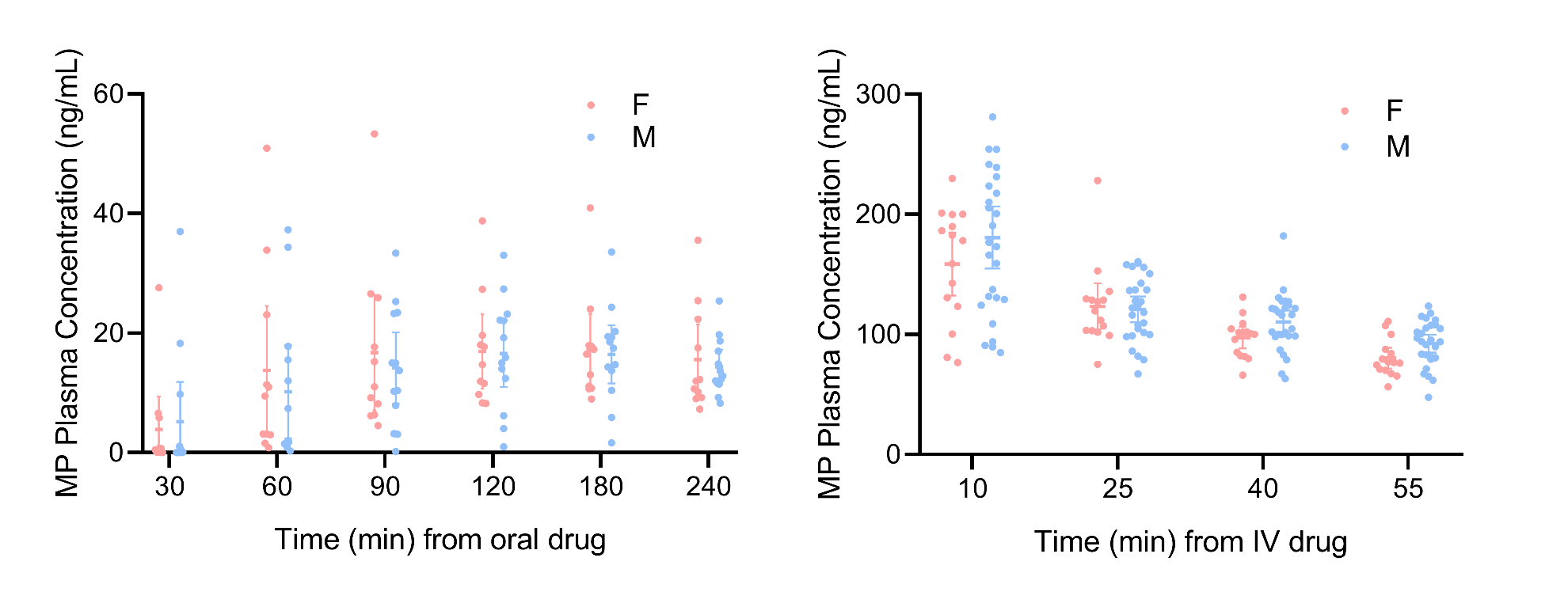


**Figure S3**. Scatter plots depicting the association between plasma concentrations of sex hormones with methylphenidate-induced dopamine release in nucleus accumbens (NAc) for females and males. The bottom row shows estradiol (left) and the progresterone:estradiol ratio (right), for which we had a lower sample size of n = 17 (seven female) participants, due to insufficient plasma sample volume. None of these sex hormones had a significant association with NAc dopamine release; see main manuscript for statistical analyses. Note: FSH = Follicular Stimulating Hormone.


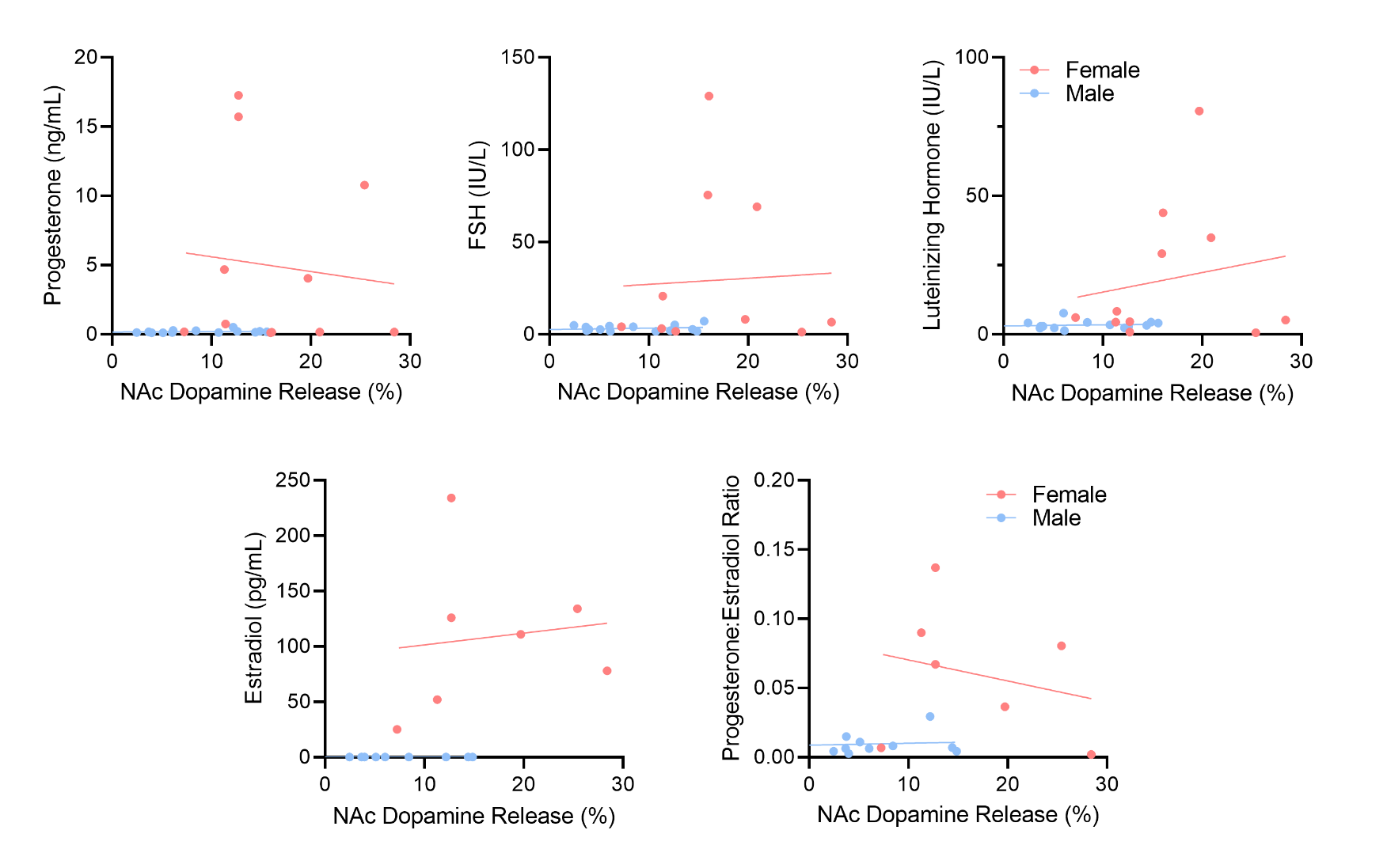

Supplement: Supplementary file 1 — Supplement [file 41380_2021_1294_MOESM1_ESM.docx]
